# Excision of Upper Jaw; Recovery

**Published:** 1872-08

**Authors:** 


### ARTICLE VI.

### *Excision of Upper Jaw; Recovery.*

(Under the care of Mr. Erichsen.)

D. K.—, a farmer, aged twenty-nine, was admitted into the hospital on Feb. 6th, 1872. Has been a strong, healthy man and has had no serious illness. About the beginning of last August he began to have a feeling of numbness over the left cheek, radiating from a point about midway between the external angle of the mouth and the external canthus. When he gaped he had a feeling of tension as of a cord through the cheek, and also a tickling sensation along the ridge forming the inner boundary of the cheek. There has at no time been pain or throbbing. About the same time he began to notice a projection downwards from the roof of the mouth. Shortly after this a small swelling appeared on the spot above referred to. This appears to have increased upwards rather rapidly, so that in September his friends noticed that it was encroaching upon his eye by pushing upwards the lower eyelid. It also pushed up the inner canthus and pressed upon the lachrymal sac. There was epiphora. Five years ago an abscess formed over the lachrymal sac, from which pus and a watery fluid have been discharged up to the present time. Soon after the eye was affected the swelling began to press upon the nose, so as ultimately to prevent his breathing through the left nostril. Epistaxis, but never excessive, has occasionally occurred. As the tumore increased the upper molars become loose and were removed, as also the pre-molars, except a part of the first; from the cavities left there has at times been a little bleeding. During the last two months his friends think he has lost flesh a little.

*State on Admission.*—An oblong tumor occupies the inner part of the left cheek. It measures two inches and three-quarters from above downwards, and two inches and a quarter from outside to inside. It has a defined edge on the outer side corresponding with a vertical line through the outer canthus. Below it extends to within half an inch of the left angle of the mouth. On the inner side it has a defined edge where it encroaches upon the nose. Above, it repsses up into the orbid, especially on the inner side. The tumor is not at all tender to tolerable pressure; it feels elastic throughout; no crackling; it is semi-fluctuating where it approaches the eye and nose. The skin is not adherent; color natural. The left nostril is closed by the pressure of the tumor. The eye is ordinarily closed, the lower lid being pushed up; when it is pushed down the patient can read without much difficulty. The pupil contracts perfectly; the conjunctive is slightly injected; the caruncular protudes more than on the right side, On looking into the mouth a tense oval mass is seen protuding from the roof. It is confined to the left half of the palate, and extends as far back as the last molar. Like the external mass, it does not crackle on pressure, but feels very elastic; the mucous membrane covering it is very pale. No enlarged glands in the submaxillary region.

On Febuary 7th, the patient being under chloroform, Mr. Erichsen performed the following operation:—The left median incisor was first extracted; then two ring forceps, with elastic bands around them to keep them tightly closed, were introduced just inside the angle of the mouth. These compressed the coronary and other branches of the facial, and thus prevented hemorrhage during the operation. An incision was made a little to the left of the middle line around the ala nasi, and along the side of the nose to just below the inner canthus, then outwards over the malar bone, nearly as far as the articulation of the lower jaw. This triangular flap was then thrown downwards and outwards. Mr. Erichsen then sawed through the hard palate from the mouth. The nasal process of the maxilla, lachrymal bone, &c., were broken through by a curved pair of bone forceps, thus opening into the bony orbit. The malar bone was then sawed through. As the mass was too soft to apply the lion forceps, Mr. Erichsen gradually extricated it with his fingers, cutting through the soft palate with a pair of long curved scissors. Two plugs of lint were stuffed into the wound, the coronary and one or two small branches tied, the ligatures cut close, and the flap was brought together by silver sutures and harelip pins at inner canthus and upper lip.

*Description of Tumor.*—The mass filled up the antrum, and Mr. Erichsen thought went back as far as the sphenoidal cells. There were a few thin scattered bits of bone on the anterior surface, and also on the side towards the nose. On the posterior aspect the bone was quite gone. Its general consistency was something between gelatinous and pulpy; color slightly pinkish. Section of the mass hardened in spirit showed bundles of fibrous tissue, but not arranged so as to form a cancerous stroma; a number of simple-rounded cells, and mass of spindle-shaped cells.—*London Lancet.*

